# The role of serum magnesium in the prediction of acute kidney injury after total aortic arch replacement: A prospective observational study

**DOI:** 10.5937/jomb0-48779

**Published:** 2024-06-15

**Authors:** Xinyi Jiang, Ziyun Li, Chixing Pan, Heng Fang, Wang Xu, Zeling Chen, Junjiang Zhu, Linling He, Miaoxian Fang, Chunbo Chen

**Affiliations:** 1 South China University of Technology, School of Medicine, Guangzhou, Guangdong Province, China; 2 Southern Medical University, Guangdong Provincial People's Hospital (Guangdong Academy of Medical Sciences), Department of Intensive Care Unit of Cardiac Surgery, Guangzhou, Guangdong Province, China; 3 Guangdong Medical University, Maoming Clinical College, Maoming, Guangdong Province, China; 4 Southern Medical University, Guangdong Provincial People's Hospital (Guangdong Academy of Medical Sciences), Department of Critical Care Medicine, Guangzhou, Guangdong Province, China; 5 Shenzhen People's Hospital, Department of Critical Care Medicine, Shenzhen, Guangdong Province, China; 6 Southern Medical University, Guangdong Provincial People's Hospital (Guangdong Academy of Medical Sciences), Department of Intensive Care Unit of Cardiac Surgery, Guangzhou, Guangdong Province, China + Shenzhen People's Hospital, Department of Critical Care Medicine, Shenzhen, Guangdong Province, China

**Keywords:** acute kidney injury, serum magnesium, cardiovascular surgical intensive care unit, total aortic arch replacement, acute aortic dissection, akutna povreda bubrega, magnezijum u serumu, kardiovaskularna hirurška jedinica intenzivne nege, totalna nadoknada luka aorte, akutna disekcija aorte

## Abstract

**Background:**

Considerable morbidity and death are associated with acute kidney damage (AKI) following total aortic arch replacement (TAAR). The relationship between AKI following TAAR and serum magnesium levels remains unknown. The intention of this research was to access the predictive value of serum magnesium levels on admission to the Cardiovascular Surgical Intensive Care Unit (CSICU) for AKI in patients receiving TAAR.

**Methods:**

From May 2018 to January 2020, a prospective, observational study was performed in the Guangdong Provincial People's Hospital CSICU. Patients accepting TAAR admitted to the CSICU were studied. The Kidney Disease: Improving Global Outcomes (KDIGO) definition of serum creatinine was used to define AKI, and KDIGO stages two or three were used to characterize severe AKI. Multivariable logistic regression and area under the curve receiver-operator characteristic curve (AUC-ROC) analysis were conducted to assess the predictive capability of the serum magnesium for AKI detection. Finally, the prediction model for AKI was established and internally validated.

**Results:**

Of the 396 enrolled patients, AKI occurred in 315 (79.5%) patients, including 154 (38.8%) patients with severe AKI. Serum magnesium levels were independently related to the postoperative AKI and severe AKI (both, P < 0.001), and AUC-ROCs for predicting AKI and severe AKI were 0.707 and 0.695, respectively. Across increasing quartiles of serum magnesium, the multivariable-adjusted odds ratios (95% confidence intervals) of postoperative AKI were 1.00 (reference), 1.04 (0.50-2.82), 1.20 (0.56-2.56), and 6.19 (2.02-23.91) (P for Trend < 0.001). When serum magnesium was included to a baseline model with established risk factors, AUC-ROC (0.833 vs 0.808, P = 0.050), reclassification (P < 0.001), and discrimination (P = 0.002) were further improved.

**Conclusions:**

Serum magnesium levels on admission are an independent predictor of AKI. In TAAR patients, elevated serum magnesium levels were linked to an increased risk of AKI. In addition, the established risk factor model for AKI can be considerably improved by the addition of serum magnesium in TAAR patients hospitalized in the CSICU.

## Abstract (sr)

**Uvod:**

Značajan morbiditet i smrt su povezani sa akutnim oštećenjem bubrega (AKI) nakon totalne zamene aortnog luka (TAAR). Veza između AKI nakon TAAR-a i nivoa magnezijuma u serumu ostaje nepoznata. Namera ovog istraživanja bila je da se pristupi prediktivnoj vrednosti nivoa magnezijuma u serumu pri prijemu u Jedinicu intenzivne kardiovaskularne hirurgije (CSICU) za AKI kod pacijenata koji primaju TAAR.

**Metode:**

Od maja 2018. do januara 2020. sprovedena je prospektivna opservaciona studija u narodnoj bolnici provincije Guangdong CSICU. Proučavani su pacijenti koji prihvataju TAAR primljeni u CSICU. Bolest bubrega: poboljšanje globalnih ishoda (KDIGO) definicija serumskog kreatinina je korišćena za definisanje AKI, a KDIGO faze dva ili tri su korišćene za karakterizaciju teške AKI. Analiza multivarijabilne logističke regresije i površine ispod krive karakteristične krive prijemnik-operater (AUC-ROC) je sprovedena da bi se procenila prediktivna sposobnost serumskog magnezijuma za detekciju AKI. Konačno, model predviđanja za AKI je uspostavljen i interno potvrđen.

**Rezultati:**

Od 396 uključenih pacijenata, AKI se javio kod 315 (79,5%) pacijenata, uključujući 154 (38,8%) pacijenata sa teškim AKI. Nivoi magnezijuma u serumu su bili nezavisno povezani sa postoperativnim AKI i teškim AKI (oba, P < 0,001), a AUC-ROC za predviđanje AKI i teškog AKI su bili 0,707 odnosno 0,695. U rastućim kvartilima serumskog magnezijuma, multivarijabilno prilagođeni odnosi šansi (95% intervala poverenja) postoperativnog AKI su bili 1,00 (referenca), 1,04 (0,50-2,82), 1,20 (0,56-2,56) i 6,19-232. P za Trend < 0,001). Kada je magnezijum u serumu uključen u osnovni model sa utvrđenim faktorima rizika, AUC-ROC (0,833 prema 0,808, P = 0,050), reklasifikacija (P < 0,001) i diskriminacija (P = 0,002) su dodatno poboljšani.

**Zaključak:**

Nivoi magnezijuma u serumu pri prijemu su nezavisni prediktor AKI. Kod pacijenata sa TAAR, povišeni nivoi magnezijuma u serumu bili su povezani sa povećanim rizikom od AKI. Pored toga, uspostavljeni model faktora rizika za AKI može se značajno poboljšati dodatkom serumskog magnezijuma kod pacijenata sa TAAR hospitalizovanim u CSICU.

## Introduction

Acute aortic dissection (AAD) is a fatal condition marked by substantial morbidity and mortality, particularly in type A aortic dissection (TAAD) [1]. The most complex and challenging surgical method used to treat TAAD is total aortic arch replacement (TAAR) [2]. Nearly all major surgical operations have the inescapable hazards of systemic complications and end-organ damage, and aortic arch surgery is especially pertinent [3] [4]. Longer cardiopulmonary bypass (CPB) and deep hypothermic circulatory arrest (DHCA) are used during the complicated open TAAR procedure, including aortic stent placement and aortic remodeling, which alters the blood supply to end organs and eventually increases renal ischemia-reperfusion injury (IRI) [5] [6] [7]. In comparison to other cardiac procedures, acute kidney injury (AKI), a prevalent postoperative complication especially after aortic surgery, carries a risk of up to 71.94% [8]. Cardiac surgery-associated AKI (CSA-AKI) has a complicated pathogenesis involving IRI, inflammatory response, metabolic imbalance, oxidative stress, and other factors [9] [10] [11] [12]. Since there is presently no efficient treatment for CSA-AKI, meaningful prognostic predictors could be able to assist clinicians in making an early diagnosis and stratifying patients by the most effective and timely intervention.

The second most abundant intracellular cation is magnesium, which is involved in several physiological processes connected to osteoporosis and cardiovascular function, including vascular tone, endothelial function, glucose and insulin metabolism [13]. Renal excretion is the key regulating element in magnesium homeostasis, which is commonly referred to as a proper equilibrium between dietary magnesium intake and excretion [14]. In people with good renal function, tubular reabsorption and excretion through urine regulate magnesium homeostasis [15]. Recently, it was discovered that renal function and serum magnesium levels are related. For example, low serum magnesium levels may increase the potential for kidney function deterioration among chronic kidney disease (CKD) patients [16]. Additionally, impaired renal function might result in the disruption of magnesium excretion [17]. CKD patients have an increased risk for chronic hypermagnesemia, which is defined by a standard dialysate magnesium level greater than 1.20 mmol/L [10] [18]. Moreover, patients who had early elevated serum magnesium levels following cardiac surgery had a considerably higher risk of AKI, according to a recent multicenter retrospective study [19]. The aforementioned results serve as a reminder that more investigation is required to elucidate any potential causal connections between serum magnesium and AKI.

In order to evaluate the effectiveness of serum magnesium on CSICU admission in predicting AKI following TAAR, we implemented a prospective, observational study.

## Materials and methods

### Patients

This prospective investigation was carried out at the Department of Cardiovascular Surgery Intensive Care Unit (CSICU), Guangdong Provincial People’s Hospital (Guangzhou, China). We enrolled TAAR patients admitted to the CSICU from May 2018 to January 2020 as determined by the attending physician. Independent doctors blinded to serum magnesium levels were free to choose the appropriate course of clinical treatment. The following were the exclusion standards: (1) under 18 years of age, (2) renal transplantation or nephrectomy, (3) renal replacement therapy (RRT) or end-stage renal disease (ESRD) before CSICU admission, (4) length of CSICU stay being less than 24 h, (5) lack of admission clinical data, and (6) refusal to consent. The criteria for ESRD were a baseline estimated glomerular filtration rate (eGFR) of less than 15 mL/min/1.73 m^2^ or receiving hemodialysis. This study was approved by the Ethics Committee of the Guangdong Provincial People’s Hospital and was conducted in accordance with the Declaration of Helsinki. In formed consent papers were signed by each participant.

### Data sources

Patients with TAAR who needed CSICU hospitalization were eligible for enrollment. Only the first surgical hospitalization indices were included when patients underwent multiple cardiovascular surgeries. Patient demographic information, laboratory parameters and clinical data were prospectively obtained via the hospital’s electronic medical record system. The participant factors that were included were as follows: age, sex, body mass index, preexisting comorbidities such as hypertension, stroke, diabetes, coronary artery disease, previous cardiac surgery and New York Heart Association (NYHA) functional class. Pre-operative two-dimensional echocardiographic data calculated by the modified Simpson method included ascending aorta (AA) diameter, left ventricular end-diastolic diameter (LVDD) and left ventricular ejection fraction (LVEF). Hemoglobin, D-dimer, hematocrit, albumin, and baseline estimated glomerular filtration rate (eGFR), which was calculated by the CKD-Epidemiology Collaboration (EPI) creatinine equation [20]. Surgical data were included as follows: American Society of Anesthesiologists (ASA) degree, specific surgery type based on TAAR including valvular surgery and coronary artery bypass grafting (CABG), emergent surgery, the use of drugs including diuretics and vasopressor drugs, CPB time, aortic cross-clamping (ACC) time, DHCA time, intraoperative intra-aortic balloon pump (IABP) support, operation time, fluid management of crystalloid and colloid, transfusion of packed red blood cells and blood platelets, and the use of magnesium supplementation. The sequential Organ Failure Assessment (SOFA) score, serum magnesium and total bilirubin levels were recorded immediately after admission. SOFA scoring was used to estimate the overall condition of patients and was evaluated 30 minutes after recovery from anesthesia.

### Exposure measurement

The principal exposure variable in this investigation was total serum magnesium which likely represents the bioactive form of magnesium. Within an hour of CSICU admission, blood samples were taken using heparin tubes. Following collection and storage, all of the samples from the participating department of the hospital were subjected to batch analysis within 24 h. The Beckman Coulter AU5800 automatic analyzer was used to analyze samples with the dimethyl aniline blue colorimetric technique. Additional variables, such as intraoperative use of magnesium supplementation that may influence exposure were recorded.

### Definitions and outcomes

The Kidney Disease: Improving Global Out comes (KDIGO) criteria was used to assess the primary outcome, which was the diagnosis of AKI, defined as a raise in baseline sCr of 26.5 μmol/L (0.3 mg/dL) in 48 hours or an increase of 50% in sCr from the preoperative level in 7 days [21]. The baseline sCr level was defined as the most recent measured sCr value within 7 days before surgery. After CSICU admission, we repeatedly measured SCr values for 7 consecutive days. The highest sCr value was determined when multiple sCr levels were measured on the same day. The urine output criterion (< 0.5 mL/kg/h for 6 h) was not used to define AKI because diuretics and intravascular hypovolemia, which are both common in patients undergoing cardiac surgery, could cause confounding [22].

Severe AKI, defined as KDIGO stage 2 or stage 3 within 7 days following CSICU admission, was the secondary endpoint. According to KDIGO’s sCr criterion, AKI is categorized as follows: In the first stage, postoperative sCr increases to 1.5–1.9 times baseline, or ≥26.5 μmol/L (≥0.3 mg/dL); in the second, sCr increases to 2.0–2.9 times baseline; in the third, sCr increases to three times baseline, or ≥353.6 μmol/L (≥4.0 mg/dL) from baseline level, or the start of RRT. RRT types included continuous kidney replacement therapy, intermittent hemodialysis and continuous ambulatory peritoneal dialysis. The criteria of CKD were eGFR less than 60 mL/min/1.73 m^2^.

### Statistical analyses

Software R 4.3.1 and SPSS 26.0 were utilized to process all statistical data. First, we divided all TAAR patients into non-AKI and AKI groups. Secondly, in order to assess clinical stepwise correlations, the admission serum magnesium levels were divided into quartiles (0.56–0.91, 0.92–1.02, 1.03–1.24, and 1.25–2.31 mmol/L for quartiles 1–4, respectively). Measured variables with a normally distributed are represented as the mean ± standard deviation, while non-normally distributed continuous data are displayed as medians and interquartile ranges. To compare continuous variables, the ANOVA test, the Kruskal-Wallis test, or Cuzick’s test for trend were performed. To compare the intergroup differences in categorical variables presented as frequencies (%), the chi-square test was used. The odds ratio (OR) and 95% confidence interval (CI) of each factor were calculated using maximum likelihood estimation in logistic regression analysis. In order to evaluate the relationship between clinical outcomes and the serum magnesium quartile as a continuous variable or a quartile-level categorical variable, serum magnesium level was classified on demand and then logistic regression was performed.

The multivariate logistic regression analysis included the following factors (*P* < 0.05) that had statistical significance in the univariate analysis to identify independent predictors and build the AKI prediction model (Model 1): age, stroke and hypertension history, preoperative LVDD and D-dimer, baseline eGFR, ASA ≥3, CPB time during the surgery, and total bilirubin on admission and SOFA score after admission. Then we calculated the adjusted ORs with random intercepts. The chosen biomarker, serum magnesium, was added into Model 1 and then Model 2 was generated. Finally, we compared Models 1 and 2 to assess the performance of predictive improvement via the DeLong test for detecting differences in the area under the curve receiver-operator characteristic curve (AUC-ROC), net reclassification improvement (NRI) index, and integrated discrimination improvement (IDI) index [23]. The average improvement in the anticipated probability of AKI was displayed by IDI, and NRI performed as a correlated indicator of the quantity of patients whose anticipated likelihood of AKI was increased. The calibration of models is shown by calibration curves in terms of the conformity between the actual information and AKI diagnosis predictions [24]. In addition, a novel technique called decision curve analysis (DCA) has been developed to assess prediction models, which aimed to acquire the clinical utility and usefulness of the suggested competing risk model [25]. Finally, we conducted a method by randomly dividing the database into a set for training (70%) and a set for validation (30%) to take independent observations to generate AUC-ROC for internal validation. The performance of the models for AKI detection was verified using each of the aforementioned internal verification techniques with 100 replications. In every test, a two-tailed *P* value less than 0.05 was deemed statistically significant.

## Results

### Cohort characteristics

**Figure 1:**
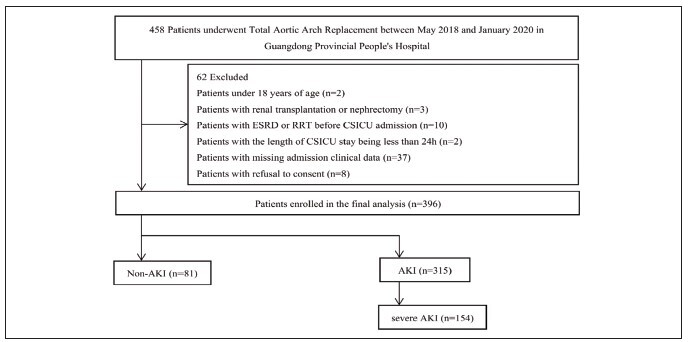
Population flowchart for the study. ESRD end-stage renal disease, RRT renal replacement therapy, AKI acute kidney injury, CSICU cardiovascular surgical intensive care unit.

Between May 2018 and January 2020, 458 patients getting TAAR were enrolled. 396 patients had been included in the final analysis after excluding those under 18 years old (n =2), those with preexisting impaired kidney function such as renal transplantation or nephrectomy (n =3), ESRD or RRT before CSICU admission (n =10) and an CSICU stay length being less than 24 h (n =2), missing admission clinical data (n =37) and those who refusal to consent (n =8). Utilizing the sCr diagnostic criteria of KDIGO, patients were classified as AKI and Non-AKI groups. During the observation period of 7 consecutive days, 315 (79.5%) patients developed AKI, and 154 (38.8%) developed severe AKI during hospitalization (Figure 1). Three of patients with severe AKI had subsequently died in the hospital.

Table 1 displays the baseline clinical features and results for the two groups. In contrast to patients without AKI, individuals with AKI were older, had more serious comorbid conditions such as hypertension, higher preoperative D-dimer, worse preoperative cardiac function involving a larger LVDD and a narrower AA diameter and a worse basic kidney function including lower eGFR level, severe general condition such as ASA ≥3 degree, and longer CPB time during the surgery. After CSICU admission, higher SOFA scores and serum magnesium levels were observed in the AKI group. Nonetheless, Nonetheless, there was no discernible variation in the intraoperative magnesium supplement consumption between the two groups (*P* = 0.320), suggesting that there was no meaningful impact of magnesium supplementation on the key outcomes.

**Table 1:** Study population baseline characteristics according to AKI. Categorical variables are shown as number (%). Continuous variables are shown as means (± standard deviation) or median (25th–75th percentile). AA ascending aorta, NYHA New York heart association, LVDD left ventricular end-diastolic diameter, LVEF left ventricular ejection fraction, ASA American Society of Anesthesiologists, ACC aortic cross-clamping, CPB cardiopulmonary bypass, CABG coronary artery bypass grafting, IABP intra-aortic balloon pump, eGFR creatinine-based estimated glomerular filtration rate, SOFA sequential organ failure assessment, CSICU cardiovascular surgical intensive care unit, AKI acute kidney injury.

Characteristic	Total	AKI	Non-AKI	P Value
No. of patients	396	315	81	
Age, year	51.6 (11.5)	52.8 (10.8)	47.3 (12.9)	< 0.001
Body mass index, kg/m^2^	24.3 (4.6)	23.7 (3.9)	24.4 (4.8)	0.199
Male sex, n (%)	326 (82.3)	286 (81.2)	70 (86.4)	0.280
Comorbidities, n (%)				
Stroke	374 (94.4)	294 (93.3)	80 (98.8)	0.103
Hypertension	209 (52.8)	175 (55.6)	34 (42.0)	0.040
Diabetes	15 (3.8)	13 (4.1)	2 (2.5)	0.711
Coronary artery disease	13.3 (3.3)	11 (3.5)	2 (2.5)	0.911
Previous cardiac surgery	357 (90.2)	283 (89.8)	74 (91.4)	0.842
Preoperative cardiovascular status				
NYHA ≥3, n (%)	311 (78.5)	247 (78.4)	64 (79.0)	1.000
LVEF, %	63.0 (8.0)	63.4 (7.6)	61.1 (59.4)	0.053
LVDD, mm	49.6 (8.4)	51.9 (10.8)	49.0 (7.5)	0.005
AA diameter, mm	43.2 (8.65)	45.1 (11.8)	52.8 (10.8)	0.030
ASA ≥3, n (%)	360 (90.9)	292 (92.7)	68 (84.4)	0.026
Preoperative laboratory tests				
Hemoglobin, g/L	120.3 (20.2)	123.2 (18.8)	119.6 (20.5)	0.144
D-dimer, ng/mL	4230.0 (1790.0–13392.0)	4860.0 (2030.0–15950.0)	2720.0 (1010.0–6740.0)	< 0.001
Hematocrit, %	0.4 (0.1)	0.4 (0.1)	0.4 (0.1)	0.104
Albumin, g/L	37.1 (4.1)	37.0 (2.1)	37.4 (4.3)	0.0471
Baseline eGFR, mL/min/1.73 m^2^	73.6 (30.6)	69.5 (28.7)	90.8 (32.4)	0.001
Intraoperative clinical factors				
Surgery type				
Valve(s), n (%)	294 (87.5)	234 (73.4)	60 (77.9)	0.500
CABG, n (%)	30 (7.6)	27 (8.6)	3 (3.7)	0.215
CABG + Valve (s), n (%)	28 (7.1)	25 (7.8)	3 (3.9)	0.335
Emergent surgery, n (%)	155 (39.1)	122 (38.7)	33 (40.7)	0.839
Use of drugs, n (%)				
Norepinephrine use	54 (13.7)	45 (14.1)	9 (11.8)	0.741
Adrenaline use	326 (82.5)	264 (82.8)	62 (81.6)	0.940
Diuretic use	58 (14.6)	44 (13.8)	14 (18.2)	0.425
IABP, n (%)	389 (98.2)	310 (98.4)	79 (97.5)	0.650
CPB time, min	246.0 (63.3)	250.7 (65.1)	227.7 (52.2)	0.004
ACC time, min	130.9 (44.3)	132.1 (45.6)	126.2 (38.6)	0.283
Operation time, min	439.5 (116.4)	444.2 (122.4)	420.9 (87.5)	0.108
Fluid management				
Crystalloid, mL	232.2 (349.8)	225.1 (351.7)	259.6 (343.2)	0.429
Colloid, mL	675.8 (482.0)	658.9 (443.8)	741.6 (607.3)	0.169
Packed red blood cells, u	3.3 (10.6)	3.8 (11.8)	1.69 (2.45)	0.121
Blood platelets, u	2.1 (3.0)	2.0 (2.8)	2.3 (3.8)	0.458
Magnesiumsupplementation, n (%)	40 (12.5)	11 (18.0)	29 (11.2)	0.320
Laboratory tests on CSICU admission				
Total bilirubin, μmol/	29.3 (28.2)	40.7 (29.0)	34.0 (24.0)	0.057
SOFA score	15.3 (1.6)	15.6 (1.5)	14.2 (1.5)	< 0.001
Serum magnesium, mmol/L	1.1 (0.3)	1.1 (0.3)	0.9 (0.2)	< 0.001
severe AKI, n (%)	154 (38.8)	154 (38.8)	0 (0)	< 0.001

### Relationship between the progression of AKI and serum magnesium levels

Table 2 displays the clinical attributes of the four groups, which is categorized by the quartiles of the serum magnesium level. Elevated serum magnesium levels connected with a history of hypertension (*P* for Trend = 0.001) and prior cardiac surgery (*P* for Trend = 0.041), lower preoperative hemoglobin (*P* for Trend = 0.019), albumin (*P* for Trend = 0.033) and baseline eGFR (*P* for Trend < 0.001), a higher rate of combination of CABG and Valve procedure based on TAAR (*P* for Trend = 0.044), longer CPB time (*P* for Trend = 0.007) and ACC time during the surgery (*P* for Trend < 0.001), increased total bilirubin level on admission (*P* for Trend < 0.001), and more severe organ injury, such as SOFA score after admission (*P* for Trend < 0.001). There was no significant difference in the intraoperative use of magnesium supplementation (*P* for Trend = 0.448) among the four groups. The AKI incidences were progressively higher in the quartile groups across gradually increased magnesium levels (*P* for Trend < 0.001). For severe AKI, similar stepwise links were seen (*P* for Trend < 0.001).

**Table 2:** Study population baseline characteristics according to the serum magnesium quartile levels. Categorical variables are shown as number (%). Continuous variables are shown as means (± standard deviation) or median (25th–75th percentile). AA ascending aorta, NYHA New York heart association, LVDD left ventricular end-diastolic diameter, LVEF left ventricular ejection fraction, ASA American Society of Anesthesiologists, ACC aortic cross-clamping, DHCA deep hypothermic circulatory arrest, CPB cardiopulmonary bypass, CABG coronary artery bypass grafting, IABP intra-aortic balloon pump, eGFR creatinine-based estimated glomerular filtration rate, SOFA sequential organ failure assessment, CSICU cardiovascular surgical intensive care unit, AKI acute kidney injury.

	Serum magnesium quartiles, mmol/L					
Characteristic	Total	Q1 (0.56–0.91)	Q2 (0.92–1.02)	Q3 (1.03–1.24)	Q4 (1.25–2.31)	P for <br>Trend
No. of patients	396	92	100	104	100	
Age, year	52.0 (11.0)	50.0 (13.0)	51.0 (10.0)	52.0 (11.0)	54.0 (11.0)	0.098
Body mass index, kg/m^2^	24.2 (4.6)	23.2 (4.0)	24.3 (6.2)	24.5 (3.8)	24.9 (4.0)	0.099
Male sex, n (%)	326 (82.3)	73 (79.3)	89 (89.0)	85 (81.7)	79 (79.0)	0.221
Comorbidities, n (%)						
Stroke	22 (5.5)	7 (7.6)	4 (4.0)	7 (6.7)	4 (4.0)	0.576
Hypertension	209 (52.7)	37 (40.2)	46 (46.0)	58 (55.8)	68 (68.0)	0.001
Diabetes	15 (3.7)	2 (2.2)	2 (2.0)	5 (4.8)	6 (6.0)	0.366
Coronary artery disease	13 (3.2)	2 (2.2)	2 (2.0)	3 (2.9)	6 (6.0)	0.354
Previous cardiac surgery	39 (9.8)	16 (17.4)	9 (9.0)	7 (6.7)	7 (7.0)	0.041
Preoperative cardiovascular status						
NYHA ≥3, n (%)	85 (21.4)	18 (19.6)	20 (20.0)	20 (19.2)	26 (26.0)	0.610
LVEF, %	63.0 (8.0)	63.4 (9.1)	92.9 (7.4)	62.5 (7.3)	63.3 (8.3)	0.850
LVDD, mm	49.6 (8.4)	51.3 (9.7)	49.0 (8.3)	48.6 (7.7)	49.4 (7.6)	0.113
AA diameter, mm	44.0 (9.5)	45.4 (11.3)	43.0 (8.9)	43.7 (10.6)	44.0 (6.8)	0.477
Preoperative laboratory tests						
Hemoglobin, g/L	120.3 (20.1)	123.6 (20.3)	122.6 (18.4)	119.9 (21.3)	115.4 (19.9)	0.019
D-dimer, ng/mL	4230.0<br>(1790.0–13340.0)	2925.0 <br>(1250.0–10605.0)	4580.0 <br>(1862.5-12937.5)	4195.0 <br>(1792.5–14025.0)	4870.0 <br>(2310.0–18310.0)	0.119
Hematocrit, %	35.9 (6.0)	36.9 (5.6)	35.8 (5.6)	36.3 (6.7)	34.7 (5.9)	0.074
Albumin, g/L	37.1 (4.2)	37.6 (3.8)	36.3 (4.4)	37.9 (4.3)	36.8 (4.0)	0.033
Baseline eGFR, mL/min/1.73 m^2^	73.6 (30.6)	85.4 (34.2)	81.4 (30.0)	72.0 (26,7)	56.9 (23.3)	< 0.001
ASA ≥3, n (%)	360 (90.6)	81 (88.0)	95 (95.0)	96 (92.3)	88 (88.0)	0.240
Intraoperative Factors						
CABG + Valve(s), n (%)	28 (7.0)	4 (4.3)	9 (9.0)	3 (2.9)	12 (12.0)	0.044
Emergent surgery, n (%)	241 (60.8)	31 (33.7)	39 (39.0)	43 (41.3)	42 (42.0)	0.637
CPB time, min	235.5<br>(201.0–281.0)	230.0 <br>(192.5–277.0)	229.5 <br>(202.0–273.8)	232.5 <br>(201.5–273.5)	250.0 <br>(213.0–300.8)	0.007
ACC time, min	130.9 (44.3)	128.4 (42.5)	128.7 (40.9)	121.5 (36.7)	145.2 (52.7)	0.001
DHCA time, min	19.6 (9.7)	19.4 (8.4)	18.6 (7.4)	21.3 (13.0)	19.0 (8.7)	0.202
IABP, n (%)	5 (1.2)	2 (2.2)	0 (0.0)	2 (1.9)	1 (1.0)	0.514
Operation time, min	437.5 (124.7)	447.1 (112.1)	431.8 (130.1)	433.6 (125.3)	438.5 (131.0)	0.836
Magnesium supplementation, n<br>(%)	51 (12.8)	13 (14.1)	9 (9.0)	17 (16.3)	12 (12.0)	0.448
Laboratory tests on CSICU admission						
Total bilirubin, μmol/L	31.9 (19.1–51.4)	28.1 (17.8–45.0)	31.6 (17.6–51.3)	30.7 (18.7–46.8)	42.0 (21.2–70.3)	0.001
SOFA score	15.3 (1.6)	14.7 (1.6)	14.8 (1.5)	15.4 (1.5)	16.0 (1.4)	< 0.001
Primary outcome						
AKI, n (%)	315 (79.5)	62 (67.4)	73 (73.0)	84 (80.8)	96 (96.0)	< 0.001
Secondary outcome						
severe AKI, n (%)	154 (38.8)	21 (13.6)	26 (16.8)	47 (29.8)	60 (39.6)	<

**Figure 2:**
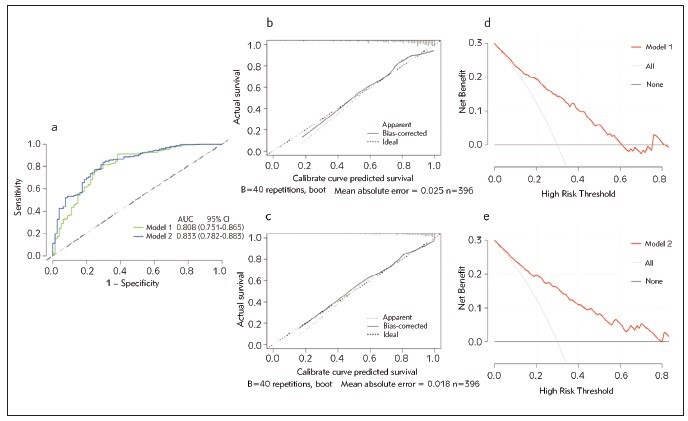
The correlation between serum magnesium and postoperative outcomes. The incidence of AKI and severe AKI was related to the quartile of serum magnesium levels (a). The AUC-ROC analysis of serum magnesium to predict postoperative AKI and severe AKI (b). AKI acute kidney injury, AUC-ROC area under the curve receiver-operator characteristic curve.

According to the histogram picture in Figure 2, the prevalence of AKI and severe AKI increased progressively as the serum magnesium levels increased (both, *P* for Trend < 0.001). For the purpose of predicting AKI and severe AKI, the individual serum magnesium AUC-ROC values were 0.707 (95% CI, 0.650–0.764) and 0.695 (95% CI, 0.641–0.748), respectively.

Higher serum magnesium levels were found to be a continuous variable linked to the development of AKI (*P* = 0.002) and severe AKI (*P* < 0.001), as Table 3 illustrates. Following multivariate adjustment, the results demonstrated that in TAAR patients, the serum magnesium level measured at admission was an independent risk indicator for AKI (*P* < 0.001) and severe AKI (*P* < 0.001). Every 1 mmol/L increase about serum magnesium was linked to a 24.53-fold increased risk of AKI (adjusted OR, 24.53; 95% CI, 4.81–149.16) and a 12.63-fold increased risk of severe AKI (adjusted OR, 12.63; 95% CI, 4.65–36.32). In addition, if serum magnesium levels were taken into account as a categorical variable, both AKI and the development of severe AKI showed a significant rising trend as the serum magnesium quartile values rose (both *P* for Trend < 0.001). After multivariate correction, patients in the highest serum magnesium group had a 6.19-fold increased risk of AKI (adjusted OR, 6.19; 95% CI, 2.02–23.91) and a 3.57-fold heightened risk of severe AKI (adjusted OR, 3.57; 95% CI, 1.79–7.30).

**Table 3:** Association between serum magnesium and postoperative outcomes. Odds ratio (95% confidence intervals) from multivariable logistic regression models were adjusted for age, stroke, hypertension history, preoperative LVDD, D-dimer, baseline eGFR, ASA ≥3, CPB time, total bilirubin on CSICU admission and SOFA score. LVDD left ventricular end-diastolic diameter, CPB cardiopulmonary bypass, ASA American Society of Anesthesiologists, SOFA sequential organ failure assessment.

		Serum magnesium		Odds ratio (95% CI)				
Outcomes	Model	OR (95% CI)	P Value	Q1 <br>(0.56–0.91, <br>mmol/L)	Q2 <br>(0.92–1.02, <br>mmol/L)	Q3 <br>(1.03–1.24, <br>mmol/L)	Q4 <br>(1.25–2.31, <br>mmol/L)	P for <br>Trend
AKI	N events/N <br>participants	315/396	N/A	62/92	73/100	84/104	96/100	N/A
	Univariable	16.23 <br>(2.72–96.87)	0.002	Reference	1.31 <br>(0.70–2.44)	2.03 <br>(1.06–3.95)	11.6 <br>(4.32–38.5)	< 0.001
	Multivariable	24.53 <br>(4.81–149.16)	< 0.001	Reference	1.04 <br>(0.50–2.82)	1.20 <br>(0.56–2.56)	6.19 <br>(2.02–23.91)	< 0.001
severe AKI	N events/N <br>participants	138/396	N/A	5/138	26/138	47/138	60/138	N/A
	Univariable	21.02 <br>(8.58–54.83)	< 0.001	Reference	1.18(0.61– <br>2.31)	2.78 <br>(1.51–5.26)	5.07 <br>(2.7–9.69)	< 0.001
	Multivariable	12.63 <br>(4.65–36.3)	< 0.001	Reference	1.12(0.62– <br>2.56)	2.36 <br>(1.22–4.67)	3.57 <br>(1.79–7.30)	< 0.001

### Relative contribution of serum magnesium to the clinical risk model for AKI

To thoroughly investigate more of the clinical contributions of adding serum magnesium to the baseline model of the established risk factors, several validation methods were performed. Established risk factors for Model 1 included age, stroke and hypertension history, preoperative LVDD and D-dimer, baseline eGFR, ASA ≥3, CPB time during the surgery, total bilirubin on admission and SOFA score after admission. Table 4 demonstrates that Model 1 was able to predict postoperative AKI with a mild AUC-ROC of 0.808 (95% CI, 0.751–0.865). After including serum magnesium in the baseline model (Model 1), Model 2 was constructed and achieved a greater AUC-ROC value of 0.833 (95% CI, 0.782–0.883), which was beyond that of Model 1. The DeLong test (*P* = 0.050) showed a statistically significant difference between the Models 1 and 2. Moreover, we observed at an extra benefit of adding serum magnesium to Model 1 for AKI prediction by using reclassification metrics. The predictive ability of Model 2 was significantly better than Model 1, as indicated by the IDI (*P* = 0.002) and NRI (*P* < 0.001) indices in patients after TAAR.

**Table 4:** Discrimination and reclassification of the combination of serum magnesium for predicting AKI. Established clinic model included age, the history of stroke and hypertension, preoperative LVDD, D-dimer, baseline eGFR, ASA ≥3, CPB time, total bilirubin and SOFA score. LVDD left ventricular end-diastolic diameter, CPB cardiopulmonary bypass, ASA American Society of Anesthesiologists, SOFA sequential organ failure assessment, AKI acute kidney injury, AUC-ROC area under the curve receiver-operator characteristic curve, CI confidence interval, NRI net reclassification improvement, and IDI integrated discrimination improvement. <br>*Estimated differences between two groups

	AUC-ROC (95%CI)	P value	NRI	P value	IDI	P value
Model 1: Established risk factor <br>model	0.808 (0.751–0.865)		Reference		Reference	
Model 2: Established risk factor <br>model + Magnesium	0.833 (0.782–0.883)	0.050*	0.47*	< 0.001	0.04*	0.002

Finally, *Figure 3a* displays the AUC-ROC curves for AKI detection using Models 1 and 2. The calibration graphs of Models 1 and 2 show a respectable level of prediction accuracy (*Figure 3b-c*). Likewise, Figure 3d-e indicates that Model 2 generated a wider range of net benefits compared to Model 1, and both models outperformed the »full treatment« or »no treatment« plans in terms of overall benefits. Furthermore, a random split of the dataset into a validation set (n = 119) and a training set (n = 277) was performed. To investigate Model 2’s internal performance, the training and validation sets were subjected to AUC-ROC analysis and the DeLong test.

The AUC-ROCs of the set for training and validation were 0.810 and 0.831, respectively (*P* = 0.810). These above results of internal validation that were repeated 100 times suggested that Model 2 has a better performance for postoperative AKI prediction.

## Discussion

These are the primary findings from our investigation. First, it was discovered that in TAAR patients, the serum magnesium level on admission was an independent predictor of the development of AKI. Second, patients with serum magnesium in the upper quartiles were strongly linked to a higher risk of AKI and severe AKI in TAAR patients. Moreover, the predictive value for AKI was much higher when serum magnesium was added to the established clinical risk model than when either biomarker or clinical risk model was used alone. Notably, this is the first prospective study to evaluate the prognostic value of serum magnesium for postoperative AKI in the TAAR cohort.

In this study, 315 (79.5%) of all 396 patients suffered AKI, and 154 (38.8%) developed severe AKI. One of the most serious and frequent side effects of cardiac surgery is AKI. Such high incidence rates of AKI correlate with difficulty in the aortic surgical setting involving prolonged intraoperative hypoxia [5]. Numerous past studies have demonstrated that the aortic procedures carry more risks, notably AKI, than other cardiac operations, such as valve replacement and CABG [26] [27].

Theoretically, AKI is a heterogeneous illness, and a combination of indicators may be more effective than a single biomarker because it might reflect multiple pathophysiological processes [28]. Our multivariate model was created after adjusting for several factors affecting the development of AKI after TAAR, which reaffirmed the fact that various types of perioperative factors triggered AKI. Our results were consistent with earlier research that found associations between postoperative AKI and the following factors: the higher ASA degree, echocardiographic parameters such as LVDD, preoperative coagulation biomarker D-dimer, operation time parameters including CPB time, and SOFA score obtained 30 minutes after recovery from anesthesia [29] [30] [31] [32]. Similarly, we found that an increased total bilirubin level upon admission increases the chance of developing CSA-AKI. This situation may be brought on by hepatic hypoperfusion for bilirubin clearance and bile transport, hemolysis, cardiotomy suction, and mechanical prosthesis during CPB [33]. More severe hyperbilirubinemia may trigger the maintenance of bile casts, causing toxicity directly to the nephrons and resulting in AKI [34]. Prediction Model 1, which was developed based on the aforementioned clinical characteristics, had an AUC-ROC value of 0.808 and did not perform better than the previously established prediction model [35] [36]. Renal tubular necrosis and the subsequent start of postoperative AKI are caused by sympathetic-adrenal medullary system overexcitation and neurohumoral regulatory dysfunction, which are more likely to be caused by hemodynamics, renal ischemia and hypoxia, systemic inflammation, and a poor general state [37] [38].

The role of serum magnesium in the development of AKI has attracted close attention worldwide. A recent study found that elevated early serum magnesium levels after surgery was independently associated with CSA-AKI [19]. In line with previous similar research, we testified that admission measurement of serum magnesium was a potential predictor for AKI following TAAR in our prospective study. Furthermore, the performance of serum magnesium on admission in AKI detection in this study had a moderate AUC-ROC value of 0.707. The data above indicate that serum magnesium holds a relatively limited predictive value when used as a single biomarker for AKI after TAAR. Interestingly, researchers discovered that the incidence of AKI was related to decrease serum magnesium levels both preoperatively and intraoperatively, and patients who received magnesium supplementation had a lower risk of AKI [39] [40]. Researchers have suggested that decreased magnesium levels have been linked to increased levels of both proatherogenic and inflammatory cytokines in endothelial cells, affecting renal function [41] [42]. However, there was essentially no difference in the intraoperative usage of magnesium supplementation through among our groups. Therefore, the effect of intraoperative magnesium supplementation on postoperative AKI requires further study by prospective intervention trials.

Moreover, several retrospective studies have found that hypermagnesemia (serum magnesium > 1.25 mmol/L) on ICU admission correlated with in-hospital death and AKI development [43] [44]. Similar to our findings, when compared to patients in the lowest quartile, the highest quartile patients had a 6.19-fold risk of AKI and a 3.57-fold risk of severe AKI. These results implied that increased serum magnesium levels after TAAR might be an alterable risk factor for AKI. Importantly, we founded that the AUC-ROC can be notably increased to 0.833 when serum magnesium is combined with the established Model 1 for AKI detection, which can reap certain clinical benefits (NRI = 0.47, IDI = 0.04) and assist in the early identification of AKI progression in clinical work. Comparing our optimized prediction model to those of other research, it exhibited comparable predictive power [45] [46].

Although the exact mechanisms by which higher magnesium levels results in AKI remain unclear, there are multiple theories regarding the magnesium metabolism pathway that underlies renal disease. Primarily, magnesium is a naturally existing calcium antagonist that is also known to boost the synthesis of local vasodilation mediators and change the way the internal vascular system reacts to different vasoactive substances [47]. More specifically, postoperative renal injury might occur from long-term hypoxia based on the cross-clamping of significant arterial structures and extracorporeal circulation during TAAR [48]. As a result, aortic surgery-induced renal blood flow reduction could drastically hamper the function of magnesium excretion, leading to elevated serum magnesium levels and this process might occur before the identification of postoperative AKI based on urine excretion or sCr criteria [44] [49] [50]. Hence, correcting magnesium homoeostasis might be a beneficial and low-cost intervention to reduce the risk of AKI.

Our study has several strengths. First, rather than concentrating only on the effects of a single biomarker, this study also evaluated the combined efficacy of serum magnesium and established multiple logistic regression prediction model in the identification of AKI. Moreover, prospective enrollment of patients helped to minimize the possibility of recollection bias. The study further includes restrictions. Initially, this study evaluated TAAR patients by a single- center studies which lacked external validation, and only Han Chinese patients were included. Second, despite the relatively large variety of factors considered, there might have been possible variables that led to the absence of results. Third, in order to dynamically determine the association between blood magnesium level and postoperative AKI, it is important to take into account the variations in serum magnesium concentration during the perioperative period at different time points. Finally, our study only recorded serum magnesium levels and supplementation with magnesium, which is not an ideal assessment of total magnesium storage in the human body. However, due to the comparative nature of this study, the above limitations do not affect the results of this study.

## Conclusion

Measurement of serum magnesium on CSICU admission is an independent predictor of AKI, and greater serum magnesium levels on admission were linked to an increased risk of AKI in TAAR patients. Finally, the established clinical risk model for AKI can be greatly enhanced by the addition of serum magnesium.

## Dodatak

### Abbreviations

AKI: Acute kidney injury;<br>AAD: Acute aortic dissection;<br>TAAD: Type A aortic dissection;<br>TAAR: Total aortic arch replacement;<br>CPB: Cardiopulmonary bypass;<br>DHCA: Deep hypothermic circulatory arrest;<br>IRI: Renal ischemia-reperfusion injury;<br>CSA-AKI: Cardiac surgery-associated acute kidney injury;<br>CKD: Chronic kidney disease;<br>CSICU: Cardiovascular surgical intensive care unit;<br>ICU: Intensive care unit;<br>ESRD: End-stage renal disease;<br>RRT: Renal replacement therapy;<br>AA: Ascending aorta;<br>NYHA: New York heart association;<br>LVDD: Left ventricular enddiastolic diameter;<br>LVEF: Left ventricular ejection fraction;<br>ASA: American Society of Anesthesiologists;<br>ACC: Aortic cross-clamping;<br>CPB Cardiopulmonary bypass;<br>CABG: Coronary artery bypass grafting;<br>IABP: Intra-aortic balloon pump;<br>sCr: Serum creatinine;<br>eGFR: Creatinine-based estimated glomerular filtration rate;<br>SOFA: Sequential organ failure assessment;<br>KDIGO: Kidney disease: improving global outcomes;<br>OR: Odds ratio;<br>CI: Confidence intervals;<br>NRI: Net reclassification improvement;<br>IDI: Integrated discrimination improvement;<br>AUC-ROC: Area under the curve receiver-operator characteristic curve;<br>DCA: Decision curve analysis.

### Declarations

### Acknowledgements

The authors appreciated all the doctors, nurses, technicians, and patients involved at Guangdong Provincial People’s Hospital for their commitment to the study.

### Ethical approval and consent to participate

This research was authorized by the Ethics Committee and executed, complying with the Declaration of Helsinki. The ethics committee of the Guangdong Provincial People’s Hospital oversaw the study (No. GDREC2015396H (R1)), covering the study design, protocol, data and sample collection, and ethical problems. Informed and written consent was obtained from either each patient or the relevant surrogates.

### Consent for publication

Not applicable.

### Availability of data and materials

The data that support the findings of this study are available upon reasonable request from the corresponding author.

### Competing Interests

The authors declare that they don’t have any known competing financial interests or personal relationships that could seem to have affected the work described in this article.

### Funding

This study was supported by the National Natural Science Foundation of China (82172162 to CBC) and the Major Program of Summit Project, Guangdong Province High-level Hospital Construction Project of Guangdong Provincial People’s Hospital (Guangdong Academy of Medical Sciences), Southern Medical University (DFJH2020028 to CBC). All funding agencies played no role in the study design, data collection, analysis, and interpretation and manuscript writing.

### Authors’ contributions

XYJ, ZYL, CXP, HF conceptualized, scheduled, and coordinated the study, wrote the manuscript, and performed data acquisition and analyses. CBC attended a significant influence on the interpretation of findings and critical scrutiny of the manuscript. LLH, MXF, JJZ supported the gathering and evaluation of the data. ZLC contributed to the sample measurement in this study. WX assisted interpret the results and examined the manuscript attentively. The final manuscript was read and approved by all authors.

### Conflict of interest statement

All the authors declare that they have no conflict of interest in this work.

## References

[1] Zhou X, Chen Z, Zhou J, Liu Y, Fan R, Sun T (2021). Transcriptome and N6-Methyladenosine RNA Methylome Analyses in Aortic Dissection and Normal Human Aorta. Front Cardiovasc Med.

[2] Song J, Wu J, Sun X, Qian X, Wei B, Wang W, et al (2021). It Is Advisable to Control the Duration of Hypothermia Circulatory Arrest During Aortic Dissection Surgery: Single-Center Experience. Front Cardiovasc Med.

[3] Tan S, Jubouri M, Mohammed I, Bashir M (2022). What Is the Long-Term Clinical Efficacy of the Thoraflex Hybrid Prosthesis for Aortic Arch Repair?. Front Cardiovasc Med.

[4] Deng Y, Yuan J, Chi R, Ye H, Zhou D, Wang S, et al (2017). The Incidence, Risk Factors and Outcomes of Postoperative Acute Kidney Injury in Neurosurgical Critically Ill Patients. Sci Rep-Uk.

[5] Wang Y, Bellomo R (2017). Cardiac surgery-associated acute kidney injury: risk factors, pathophysiology and treatment. Nat Rev Nephrol.

[6] Arnaoutakis G J, Ogami T, Patel H J, Pai C W, Woznicki E M, Brinster D R, et al (2023). Acute Kidney Injury in Patients Undergoing Surgery for Type A Acute Aortic Dissection. Ann Thorac Surg.

[7] Chang C H, Chen S W, Chen J J, Chan Y H, Yen C L, Lee T H, et al (2021). Incidence and Transition of Acute Kidney Injury, Acute Kidney Disease to Chronic Kidney Disease after Acute Type A Aortic Dissection Surgery. J Clin Med.

[8] Hobson C E, Yavas S, Segal M S, Schold J D, Tribble C G, Layon A J, et al (2009). Acute kidney injury is associated with increased long-term mortality after cardiothoracic surgery. Circulation.

[9] Xie T, Xin Q, Zhang X, Tong Y, Ren H, Liu C, et al (2022). Construction and validation of a nomogram for predicting survival in elderly patients with cardiac surgery. Front Public Health.

[10] Liu N, Li D, Liu D, Liu Y, Lei J (2023). FOSL2 participates in renal fibrosis via SGK1-mediated epithelial-mesenchymal transition of proximal tubular epithelial cells. J Transl Intern Med.

[11] Ai S, Xu L, Zheng K (2022). Acute Kidney Injury Associated with Severe Hypouricemia Caused By a Novel SLC2A9 Mutation: Enlightenment from Rare Disease to Common Disease. J Transl Intern Med.

[12] Jhaveri K D, Saratzis A N, Wanchoo R, Sarafidis P A (2017). Endovascular aneurysm repair (EVAR)-and transcatheter aortic valve replacement (TAVR)-associated acute kidney injury. Kidney Int.

[13] Volpe S L (2013). Magnesium in disease prevention and overall health. Adv Nutr.

[14] Milinković N, Zeković M, Dodevska M, Đorđević B, Radosavljević B, Ignjatović S, Ivanović N (2022). Magnesium supplementation and iron status among female students: The intervention study. J Med Biochem.

[15] Konrad M, Schlingmann K P (2014). Inherited disorders of renal hypomagnesaemia. Nephrol Dial Transplant.

[16] Van Laecke S, Nagler E V, Verbeke F, Van Biesen W, Vanholder R (2013). Hypomagnesemia and the risk of death and GFR decline in chronic kidney disease. Am J Med.

[17] Wang R, He M, Xu J (2022). Initial Serum Magnesium Level Is Associated with Mortality Risk in Traumatic Brain Injury Patients. Nutrition.

[18] Musso C G (2009). Magnesium metabolism in health and disease. Int Urol Nephrol.

[19] Xiong C, Shi S, Cao L, Wang H, Tian L, Jia Y, et al (2023). Association of early postoperative serum magnesium with acute kidney injury after cardiac surgery. Renal Failure.

[20] Levey A S, Stevens L A, Schmid C H, Zhang Y L, Castro A R, Feldman H I, et al (2009). A new equation to estimate glomerular filtration rate. Ann Intern Med.

[21] Kellum J A, Lameire N (2013). Diagnosis, evaluation, and management of acute kidney injury: a KDIGO summary (Part 1). Crit Care.

[22] Macedo E, Malhotra R, Claure-Del G R, Fedullo P, Mehta R L (2011). Defining urine output criterion for acute kidney injury in critically ill patients. Nephrol Dial Transpl.

[23] DeLong E R, DeLong D M, Clarke-Pearson D L (1988). Comparing the areas under two or more correlated receiver operating characteristic curves: a nonparametric approach. Biometrics.

[24] Cook N R (2008). Statistical evaluation of prognostic versus diagnostic models: beyond the ROC curve. Clin Chem.

[25] Vickers A J, Elkin E B (2006). Decision curve analysis: a novel method for evaluating prediction models. Med Decis Making.

[26] Lei G, Wang G, Liu Q, Zhou H, Fang Z, Zhang C, et al (2019). Single-Stage Hybrid Aortic Arch Repair is Associated With a Lower Incidence of Postoperative Acute Kidney Injury Than Conventional Aortic Surgery. J Cardiothor Vasc an.

[27] Zhou H, Wang G, Yang L, Shi S, Li J, Wang M, et al (2018). Acute Kidney Injury After Total Arch Replacement Combined With Frozen Elephant Trunk Implantation: Incidence, Risk Factors, and Outcome. J Cardiothor Vasc an.

[28] Bai Y, Li Y, Tang Z, Hu L, Jiang X, Chen J, et al (2022). Urinary proteome analysis of acute kidney injury in post-cardiac surgery patients using enrichment materials with high-resolution mass spectrometry. Front Bioeng Biotech.

[29] Fang M, Li J, Fang H, Wu J, Wu Z, He L, et al (2023). Prediction of acute kidney injury after total aortic arch replacement with serum cystatin C and urine N-acetyl-beta-d-glucosaminidase: A prospective observational study. Clin Chim Acta.

[30] Jain A, Tracci M C, Coleman D M, Cherry K J, Upchurch G J (2013). Renal malperfusion: spontaneous renal artery dissection and with aortic dissection. Semin Vasc Surg.

[31] He L, Liang S, Liang Y, Fang M, Li J, Deng J, et al (2023). Defining a postoperative mean arterial pressure threshold in association with acute kidney injury after cardiac surgery: a prospective observational study. Intern Emerg Med.

[32] Choi J S, Baek S H, Chin H J, Na K Y, Chae D W, Kim Y S, et al (2018). Systolic and diastolic dysfunction affects kidney outcomes in hospitalized patients. Bmc Nephrol.

[33] An Y, Xiao Y B, Zhong Q J (2006). Hyperbilirubinemia after extracorporeal circulation surgery: a recent and prospective study. World J Gastroentero.

[34] Patel J, Walayat S, Kalva N, Palmer-Hill S, Dhillon S (2016). Bile cast nephropathy: A case report and review of the literature. World J Gastroentero.

[35] Hou Y, Deng Y, Hu L, He L, Yao F, Wang Y, et al (2021). Assessment of 17 clinically available renal biomarkers to predict acute kidney injury in critically ill patients. J Transl Intern Med.

[36] Hu L, Gao L, Zhang D, Hou Y, He L L, Zhang H, et al (2022). The incidence, risk factors and outcomes of acute kidney injury in critically ill patients undergoing emergency surgery: a prospective observational study. Bmc Nephrol.

[37] Bagshaw S M, George C, Gibney R T, Bellomo R (2008). A multi-center evaluation of early acute kidney injury in critically ill trauma patients. Renal Failure.

[38] Luan Y, Huang E, Huang J, Yang Z, Zhou Z, Liu Y, et al (2023). Serum myoglobin modulates kidney injury via inducing ferroptosis after exertional heatstroke. J Transl Intern Med.

[39] Koh H B, Jung C Y, Kim H W, Kwon J Y, Kim N H, Kim H J, et al (2022). Preoperative Ionized Magnesium Levels and Risk of Acute Kidney Injury After Cardiac Surgery. Am J Kidney Dis.

[40] Oh T K, Oh A Y, Ryu J H, Koo B W, Lee Y J, Do S H (2019). Retrospective analysis of the association between intraoperative magnesium sulfate infusion and postoperative acute kidney injury after major laparoscopic abdominal surgery. Sci Rep-Uk.

[41] Tin A, Grams M E, Maruthur N M, Astor B C, Couper D, Mosley T H, et al (2015). Results from the Atherosclerosis Risk in Communities study suggest that low serum magnesium is associated with incident kidney disease. Kidney Int.

[42] Ferre S, Baldoli E, Leidi M, Maier J A (2010). Magnesium deficiency promotes a pro-atherogenic phenotype in cultured human endothelial cells via activation of NFkB. Biochim Biophys Acta.

[43] Laupland K B, Tabah A, Jacobs N, Ramanan M (2020). Determinants of serum magnesium abnormalities and outcome among admissions to the intensive care unit. Anaesth Crit Care Pa.

[44] Cheungpasitporn W, Thongprayoon C, Erickson S B (2015). Admission hypomagnesemia and hypermagnesemia increase the risk of acute kidney injury. Renal Failure.

[45] Qu R, Hu L, Ling Y, Hou Y, Fang H, Zhang H, et al (2020). C-reactive protein concentration as a risk predictor of mortality in intensive care unit: a multicenter, prospective, observational study. Bmc Anesthesiol.

[46] Deng J, He L, Liang Y, Hu L, Xu J, Fang H, et al (2023). Serum N-terminal pro-B-type natriuretic peptide and cystatin C for acute kidney injury detection in critically ill adults in China: a prospective, observational study. Bmj Open.

[47] Larsson S C, Burgess S, Michaelsson K (2018). Serum magnesium levels and risk of coronary artery disease: Mendelian randomisation study. Bmc Med.

[48] Novak Z, Zaky A, Spangler E L, McFarland G E, Tolwani A, Beck A W (2021). Incidence and predictors of early and delayed renal function decline after aortic aneurysm repair in the Vascular Quality Initiative database. J Vasc Surg.

[49] Morooka H, Tanaka A, Kasugai D, Ozaki M, Numaguchi A, Maruyama S (2022). Abnormal magnesium levels and their impact on death and acute kidney injury in critically ill children. Pediatr Nephrol.

[50] Galan C I, Vega A, Goicoechea M, Shabaka A, Gatius S, Abad S, et al (2021). Impact of Serum Magnesium Levels on Kidney and Cardiovascular Prognosis and Mortality in CKD Patients. J Renal Nutr.

